# Complete Genome Sequence of Salmonella enterica Serovar Heidelberg Myophage Meda

**DOI:** 10.1128/MRA.00253-19

**Published:** 2019-04-25

**Authors:** John K. Neff, Yicheng Xie, Jason J. Gill, Mei Liu

**Affiliations:** a Center for Phage Technology, Texas A&M University, College Station, Texas, USA; Loyola University Chicago

## Abstract

Salmonella enterica serovar Heidelberg is a multidrug-resistant foodborne pathogen that originated from poultry and cattle. Bacteriophages isolated for this pathogen may be used as biocontrol agents in food products or animals for preventing *Salmonella* foodborne diseases. Here, we present the complete genome sequence of *Salmonella* Heidelberg phage Meda.

## Abstract (precis)

Salmonella enterica serovar Heidelberg is a multidrug-resistant foodborne pathogen that originated from poultry and cattle. Bacteriophages isolated for this pathogen may be used as biocontrol agents in food products or animals for preventing *Salmonella* foodborne diseases.

## ANNOUNCEMENT

Salmonella enterica serovar Heidelberg is a multidrug-resistant foodborne pathogen that originated from poultry and cattle (1). As antibiotic resistance increases in foodborne pathogens, phages may be used as biocontrol agents in food or animal production to combat this rising threat.

Myophage Meda was isolated from the soil of a cattle-harvesting facility in Michigan in August 2016 by growing *Salmonella* Heidelberg in the soil extract using tryptic soy broth (Difco) at 37°C with aeration. Phages were isolated and propagated by the soft agar overlay method (2). Phage genomic DNA was prepared using a modified Promega Wizard DNA cleanup kit protocol, as described previously (3). Pooled indexed DNA libraries were prepared using the Illumina TruSeq Nano low-throughput (LT) kit, and the sequence was obtained from the Illumina MiSeq platform using the MiSeq V2 500-cycle reagent kit, following the manufacturer’s instructions, producing 312,918 paired-end reads for the index containing the phage genome. Reads were quality controlled in FastQC 0.11.5 (http://www.bioinformatics.babraham.ac.uk/projects/fastqc/), trimmed with the FASTX-Toolkit 0.0.14 (http://hannonlab.cshl.edu/fastx_toolkit/), and assembled using SPAdes 3.5.0 (4). Meda was assembled at 223.7-fold coverage. The assembled contig completion was confirmed by PCR using primers (5′-TGAGCATGGTTTCCGTTAGAG-3′ and 5′-GTGATTCTAGGCCAGTTGGTAG-3′) facing off the ends of the assembled contig and Sanger sequencing of the resulting product, with the contig sequence manually corrected to match the resulting Sanger sequencing read. GLIMMER 3.0 (5) and MetaGeneAnnotator 1.0 (6) were used to predict protein-coding genes, along with manual curation. tRNA genes were predicted using ARAGORN 2.36 (7). Protein functions were predicted using BLASTp 2.2.28 (8) to detect sequence homology. Protein structure prediction was performed using HHpred 2.1 (9), and conserved domain searches were conducted using InterProScan 5.15-5.40 (10). All analyses were done with default settings using the CPT Galaxy (11) (cpt.tamu.edu) and Web Apollo (12) interfaces.

The complete Meda genome is 84,668 bp long, consisting of 131 protein-coding genes with a coding density of 88.04%. It has a GC content of 38.85%, which is significantly lower than that of its host, *Salmonella* Heidelberg (52.08%) (13). Meda shares the most protein homology with *Salmonella* bacteriophage Felix O1 (14), with 130 similar proteins at an E value of <0.001 by BLASTp, using the GenBank nonredundant (nr) database. Like Felix O1, Meda appears not to possess a copy of RNA polymerase or any proteins homologous to known host RNA polymerase modifiers. Meda’s lysis genes are distributed across the genome rather than forming a discrete lysis cassette. While spanins and an endolysin (glycoside hydrolase) were annotated, a holin was not identified. The Meda genome contains several HNH endonucleases, one of which interrupts the large subunit of anaerobic nucleoside diphosphate reductase. Another intron containing no HNH endonuclease sequence is present in a DNA polymerase gene. A predicted polynucleotide kinase gene was identified in Meda, but the protein-coding sequence is split into two reading frames (GenBank accession numbers AXY86388 and AXY86389) by an intervening stop codon. The presence of this stop codon was confirmed by Sanger sequencing. It is not clear if this gene is essential for phage growth or if it is still functional despite this interruption.

### Data availability.

The genome sequence of phage Meda has been deposited under GenBank accession number MH586731. The associated BioProject, SRA, and BioSample accession numbers are PRJNA222858, SRR8787572, and SAMN11259651, respectively.
